# Analysis of antibiotic sensitivity and resistance genes of *Bordetella pertussis* in Chinese children

**DOI:** 10.1097/MD.0000000000024090

**Published:** 2021-01-15

**Authors:** XiaoJuan Lin, Jun Zou, Kaihu Yao, Lijun Li, Lili Zhong

**Affiliations:** a Department of Pediatrics, Hunan Provincial Key Laboratory of Pediatric Respirology, Hunan Provincial People's Hospital, Changsha; b Microbiology Laboratory of Beijing Children's Hospital, Capital Medical University, Beijing, China.

**Keywords:** *Bordetella pertussis*, drug resistance gene, drug sensitivity, erythromycin, fluorescent quantitative polymerase chain reaction, microbial culture, pertussis

## Abstract

**Objective:**

To understood the pathogen detection status and clinical characteristics of suspected pertussis in children and to observe the drug sensitivity and drug resistance genes of *Bordetella pertussis* (*B. pertussis*).

**Methods:**

Three hundred fifty-one cases were collected and their nasopharyngeal swab samples were analyzed by culture and fluorescent quantitative polymerase chain reaction. The susceptibility to erythromycin, clindamycin, ampicillin, levofloxacin, and sulfamethoxazole-trimethoprim were tested by E-test for the positive strains, and the susceptibility to erythromycin was also tested for the KB disk diffusion method. The 23S rRNA gene of the positive strains was amplified and sequenced, and statistical analysis was performed in conjunction with clinical data.

**Results:**

The positive rate of bacterial culture was 16.8% (59/351), and the positive rate of PCR was 62.4% (219/351). Two cases were positive about bacterial culture and negative for PCR. There were 221 confirmed cases of pertussis. The E-test results showed that the rate of the sensitivity of the 55 strains of pertussis to erythromycin and clindamycin was 50.9% (28/55), the minimum antibiotic concentration50 (MIC50) and MIC90 values were 0.094/>256 and 0.75/>256 mg/L, respectively, and the MIC50/MIC90 to ampicillin, levofloxacin, and sulfamethoxazole were 0.125/0.19, 0.38/0.5, and 0.125/0.25 mg/L, respectively. The KB disk diffusion method showed 27 of the 55 strains 49.1% (27/55) was resistant to erythromycin; all of the resistant strains had the 23S rRNA gene A2047G mutation, and their MIC of erythromycin was >256 mg/L.

**Conclusion:**

The diagnosis of pertussis by a fluorescent quantitative polymerase chain reaction method is more sensitive than that of bacterial culture. The resistance of *B. pertussis* to erythromycin was prominent. All of the strains of *B. pertussis* resistant to erythromycin in our center had the A2047G mutation of the 23S rRNA gene.

## 1 Introduction

Pertussis is a severe infectious disease of the respiratory tract that primarily affects infants and young children and is sometimes fatal. According to the World Health Organization (WHO) report, even though the global (DTP) vaccine coverage rate reached 86%, there were still 139,786 cases reported in 2014.^[1]^ In recent years, the issue of “resurgence of pertussis” has received extensive attention.^[2–4]^ However, in China, the incidence of whooping cough from 2010 to 2014 was 0.01264/100,000 to 0.4337/100,000, which was far lower than that in other countries.^[5]^ Chinese researchers believe that the currently available data underestimate the true situation of pertussis in China.^[6]^ The reason for this may be related to the fact that the etiological diagnosis of pertussis is not routinely carried out, and there is a lack of monitoring for pertussis and insufficient reporting of infectious diseases.

Erythromycin and other macrolide antibiotics have long been the first choice for the treatment of *Bordetella pertussis* (*B. pertussis)*. Effective antibiotic treatment can block the spread of *B. pertussis*. However, since 1994 when researchers first isolated erythromycin-resistant strains of *B. pertussis* from Yuma people in the United States,^[7]^ several countries have reported erythromycin-resistant strains.^[8,9]^ In recent years, some cities in northern China, such as Beijing, Tianjin, etc,^[10–12]^ also reported the emergence of drug-resistant *B. pertussis*. At present, the mechanism of erythromycin resistance of *B. pertussis* is mainly the A2047G mutation of the 23S rRNA gene.^[8,13]^ This mutation was found in the *B. pertussis* collected in northern China. Our previous study analyzed the pathogen in children that were suspected to have pertussis in China's southern area of Hunan Province using fluorescent quantitative polymerase chain reaction (qPCR).^[14]^ In this study, 2 methods, qPCR as well as bacterial culture, were used to detect *B. pertussis*, and then the sensitivity of *B. pertussis* to antibiotics and the A2047G mutation of the 23S rRNA gene was further analyzed.

## 2 Materials and methods

### 2.1 Research objective

From November 2017 to October 2018, children ≤14 years that were suspected of having pertussis in the Hunan Provincial People's Hospital were studied, and written informed consent was obtained from their parents. The standard for suspected pertussis refers to the “Diagnostic Standards and Treatment Principles of Pertussis” issued by the China CDC and the State Technical Supervision Bureau.^[15]^ The secretions on the nasopharyngeal swabs of the children were taken for a selective culture of *B. pertussis* and qPCR was also used to detect *B. pertussis*, and the clinical information of the children was recorded.

### 2.2 Specimen collection and testing

Specimen sampling was performed by nurses in the Children's Respiratory Department of Hunan Provincial People's Hospital. After collecting the nasopharyngeal swab, they added 2 mL of normal saline to 1 portion and stored it in a −80 °C freezer. These nasopharyngeal swab specimens were sent to the Hunan Shengxiang Biological Company for qPCR. The IS481 fragment of pertussis was selected as the target gene for amplification. The other nasopharyngeal swab was inoculated in a *B. pertussis* selection dish and immediately placed in a CO_2_ incubator (35 °C) for incubation. For colonies of suspected *B. pertussis* strains that grew, a slide agglutination test was carried out with pertussis sera and para pertussis sera. After the determination of *B. pertussis*, a single colony was selected and transferred to the *B. pertussis* basal medium and placed in a CO_2_ constant temperature incubator. After being incubated for 72 hours, the preserved strains were stored in an ultralow temperature (−80 °C) freezer and then sent on dry ice to the Microbiology Laboratory of Beijing Children's Hospital for further testing.

### 2.3 Antimicrobial sensitivity tests

The susceptibility of all positive strains to erythromycin, clindamycin, ampicillin, levofloxacin and sulfamethoxazole-trimethoprim (SMZ-TMP) were tested by the E-test. The susceptibility to erythromycin was also tested with the KB disk diffusion method. Each batch of drug susceptibility test includes the quality control strain *Staphylococcus aureu*s ATCC29213, which needs to be incubated for at least 16 hours, that is, the minimum antibiotic concentration (MIC) of antibiotics of the quality control strain *S. aureus* is read the next day. After 72 hours, the antibiotic MIC of the *B. pertussis* strain was read. The allowable range of erythromycin MIC quality control for the quality control strain *S. aureus* was implemented according to the CLSI standard.^[13]^ At present, there is no international standard for the detection methods or the results of various antibiotic resistance tests of *B. pertussis*. There are reports in the literature that for the E-test strip method, the MIC range of macrolides such as erythromycin is 32 to > 256 mg/L, which can be determined as drug-resistant.^[7–9]^ For the KB disk diffusion method, when the diameter of the bacteriostatic ring is less than 42 mm, it can be determined as erythromycin resistance.^[13,16]^

### 2.4 Detection of drug resistance genes of *B. pertussis*

Using a genomic DNA extraction kit (Beijing Cyborg Gene Technology Co., Ltd.), we extracted the genomic deoxyribonucleic acid of the isolate according to the manufacturer's instructions. The forward primers 5’-TTCCTTGTCGGGTAAGTTCC-3’ and the reverse primers 5’-GCGGTATCAGCCTGTTATCC-3’ were used.^[17]^

PCR conditions: 95 °C devaluation for 5 minutes; 95 °C denaturation for 1 minute, 60 °C annealing for 1 minute, 72 °C extension for 1 minute (35 cycles); 72 °C extension for 5 minutes; 4 °C storage for 10 minutes. The qualified product of *B. pertussis* was sent to Beijing Tianyi Huiyuan Sequencing Company for sequencing. BLAST software (http://www.ncbi.nlm.nih.gov/blast/Blast.cgi) was used to compare the determined gene sequence with the standard sequence of the erythromycin resistance gene (23S rRNA) of *B. pertussis* (GenBank sequence number: X68323)^[16]^ and we observed and recorded the mutations in this sequence.

### 2.5 Statistical analysis

SPSS19.0 statistical software was used for data processing. The measurement data conforming to the normal distribution are represented by ($\bar{\text{x}}\pm \text{s}$), the count data are represented by percentages (%), the *t* test was used for comparisons between groups, and χ2 tests were used to compare the samples rates of 2 groups. *P* < .05 was considered statistically significant.

## 3 Results

### 3.1 Positive detection of pathogen

From November 2017 to October 2018, a total of 351 nasopharyngeal swab specimens from children with suspected pertussis was collected, all of which were tested by bacterial culture and qPCR. Among them, the positive detection rate of bacterial culture was 16.8% (59/351), and the positive detection rate of qPCR was 62.4% (219/351). Two cases of bacterial culture were positive when the qPCR was negative. The number of diagnosed pertussis cases was 221, and the positive rate was 63% (221/351).

**Figure 1:**
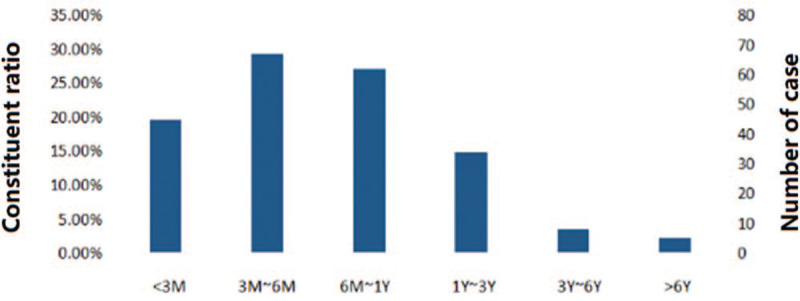
Age distribution of 221 children infected with *Bordetella pertussis*.

Among the 221 children infected with *B. pertussis*, 58.4% (129/221) were boys and 41.6% (92/221) girls; the timing of the case distribution was: spring (January–March) accounted for 16.3% (36/221), summer (April–June) accounted for 20.8% (46/221), autumn (July–September) 39.4% (87/221), and winter (October–December) 23.5% (52/221). The disease occurred throughout the year, but among the 12 months, August alone accounted for 20.4% (45/221). The minimum age of the cases was 1 month old, the maximum was 7 years old, 50.7% (112/221) were under 6 months old, 78.7% (174/221) were under 1 year old, and the majority of cases were under 3 years old, accounting for 94.1% (208/221), see Figure 1 for details. Around half (50.2% (111/221)) of the children infected with *B. pertussis* had a history of cough in their families, and 50.7% (112/221) of the 221 children had received a pertussis vaccination. Before the collection of specimens, 82.4% (182/221) of them had a history of antibiotic use, of which macrolides alone accounted for 18.7% (34/182), cephalosporin alone accounted for 36.8% (67/182), and combined 34.6% (63/182).

### 3.2 Analysis of clinical symptoms in children infected with *B. pertussis*

The 351 children with suspected pertussis were divided into the BP group and non-BP group according to the pathogen diagnosis. The incidences of spastic cough and lymphocyte ratio in the BP group were higher than those in the non-BP group. See Table 1.

**Table 1:** Comparison of clinical characteristics between children with and without *Bordetella pertussis* infection [cases (%)].

	BP group (n = 221)	Non-BP group (n = 107)	X^2^	*P*
Spastic cough	200 (90.50)	85 (79.44)	7.740	.005
Whoop	74 (33.48)	26 (24.30)	2.870	.09
Cyanosis	65 (29.41)	29 (27.10)	0.188	.665
Apnea	26 (11.76)	7 (6.54)	2.173	.14
Fever	54 (24.43)	31 (28.97)	0.773	.379
Rhinorrhea	115 (52.04)	44 (41.12)	3.439	.064
Nasal obstruction	72 (32.58)	24 (22.43)	3.587	.058
Pneumonia detected in image results	115 (52.04)	61 (57.01)	1.838	.175
inpatient d (d, $\bar{\text{x}}\pm \text{s}$)	8.61 ± 6.24	8.85 ± 5.50	24.832	.415
WBC (×10^9^/L, $\bar{\text{x}}\pm \text{s}$)	15.59 ± 8.53	10.86 ± 4.90	251.509	.247
L (%, $\bar{\text{x}}\pm \text{s}$)	67.45 ± 12.72	61.00 ± 14.60	205.140	.03

WBC = White blood cell.

The children in the BP group were further divided into the pertussis-vaccinated group and the unvaccinated group. The incidence of cyanosis in the vaccinated group was less than that in the unvaccinated group, and the difference was statistically significant (*P* < .05). For details, see Table 2.

**Table 2:** Clinical features by pertussis vaccination history [cases (%)].

	Vaccinated group (n = 112)	Unvaccinated group (n = 107)	X^2^	*P*
Spastic cough	103 (91.96)	97 (88.99)	0.568	.451
Whoop	36 (32.14)	38 (34.86)	0.183	.668
Cyanosis	25 (22.3)	40 (36.70)	5.499	.019
Apnea	9 (8.04)	17 (15.60)	3.042	.081
Fever	27 (24.11)	27 (24.77)	0.013	.909
Rhinorrhea	60 (53.57)	55 (50.46)	0.214	.643
Nasal obstruction	38 (33.93)	34 (31.19)	0.188	.664
Positive bacterial culture	27 (45.8)	32 (54.3)	0.778	.378
inpatient days (d, $\bar{\text{x}}\pm \text{s}$)	7.57 ± 3.03	9.61 ± 8.10	17.378	.564
WBC (×10^9^/L, $\bar{\text{x}}\pm \text{s}$)	15.09 ± 7.46	16.04 ± 9.42	169.996	.442
L (%, $\bar{\text{x}}\pm \text{s}$)	67.49 ± 11.51	67.42 ± 13.79	130.646	.280

WBC = White blood cell.

According to whether or not antibiotics were used before collecting the pathogen test specimens of children infected with *B. pertussis*, they were divided into the antibiotic group and the non-antibiotic group. There were no significant differences between the 2 groups in the incidences of spastic cough, whoop, apnea, or other clinical symptoms and the positive detection rate of bacterial culture (*P*>.05), see Table 3. However, the group of children infected with *B. pertussis* that used macrolide antibiotics before the collection of the pathogen test samples showed a lower positive detection rate (*P* < .05), see Table 4.

**Table 3:** Clinical characteristics of children using antibiotics before collection of the specimens [cases (%)].

	Antibiotics group (n = 182)	Non-Antibiotics group (n = 39)	X^2^	*P*
Spastic cough	167 (91.76)	33 (84.62)	1.906	.167
Whoop	62 (34.07)	12 (30.77)	0.157	.629
Cyanosis	55 (30.22)	10 (25.64)	0.324	.569
Apnea	23 (12.64)	3 (7.69)	0.757	.384
Fever	43 (23.63)	11 (28.21)	0.365	.546
Rhinorrhea	60 (53.57)	55 (50.46)	2.300	.129
Nasal obstruction	63 (34.62)	9 (23.08)	1.947	.163
Positive bacterial culture	45 (24.73)	14 (35.90)	2.048	.152

**Table 4:** Clinical characteristics of children using macrolide antibiotics before collection of the samples [cases (%)].

	Macrolide group (n = 119)	Non-Macrolide group (n = 102)	X^2^	*P*
Spastic cough	109 (91.76)	91 (89.22)	0.362	.354
Whoop	39 (32.77)	35 (34.31)	0.059	.460
Cyanosis	40 (33.61)	25 (24.51)	2.192	.091
Apnea	13 (10.92)	13 (12.75)	0,175	.416
Fever	29 (24.37)	25 (24.51)	0.001	.552
Rhinorrhea	46 (38.66)	69 (67.65)	3.654	.038
Nasal obstruction	41 (34.45)	31 (30.39)	0.412	.310
Positive bacterial culture	19 (15.97)	40 (39.21)	15.170	.000

### 3.3 Drug sensitivity of the *B. pertussis* strains

The drug sensitivity results of the 55 *B. pertussis* culture-positive strains by the E-test method showed that the MIC50 and MIC90 of the *B. pertussis* strain to erythromycin and clindamycin were 0.094/> 256 and 0.75/> 256 mg/L; 49.1% (27/55) strains had a MIC of erythromycin and clindamycin> 256 mg/L, and the remaining 28 strains had a MIC of erythromycin and clindamycin of 0.016 to 0.094 mg/L and 0.019 to 0.75 mg/L. The MIC50/MIC90 of the 55 strains for ampicillin, levofloxacin, and SMZ-TMP were 0.125/0.19, 0.38/0.5, and 0.125/0.25 mg/L, respectively. The KB disk diffusion method showed that the diameter of the erythromycin inhibitory ring of 27 cases was 6 mm, and the diameter of the erythromycin inhibitory ring of 28 cases was> 42 mm (range 48–60 mm), which corresponded to the E-test results. The results of drug resistance testing of 55 cases of *B. pertussis* strains are shown in Table 5.

**Table 5:** Test results of 55 strains of *Bordetella pertussis* (n = 55).

	E-test (mg/L)			KB disk diffusion method (mm)	
Antibiotics	MIC50	MIC90	MIC value	The diameter of the inhibitory ring	Sensitive rate
erythromycin	0.094	>256	0.016–>256	6–60^#^	50.9%
clindamycin	0.75	>256	0.019–>256		
ampicillin	0.125	0.19	0.064–0.25		
levofloxacin	0.38	0.5	0.19–0.75		
sulfamethoxazole-trimethoprim	0.125	0.25	0.023–0.38		

MIC = minimum antibiotic concentration.

### 3.4 Detection of the erythromycin resistance gene of *B. pertussis*

**Figure 2:**
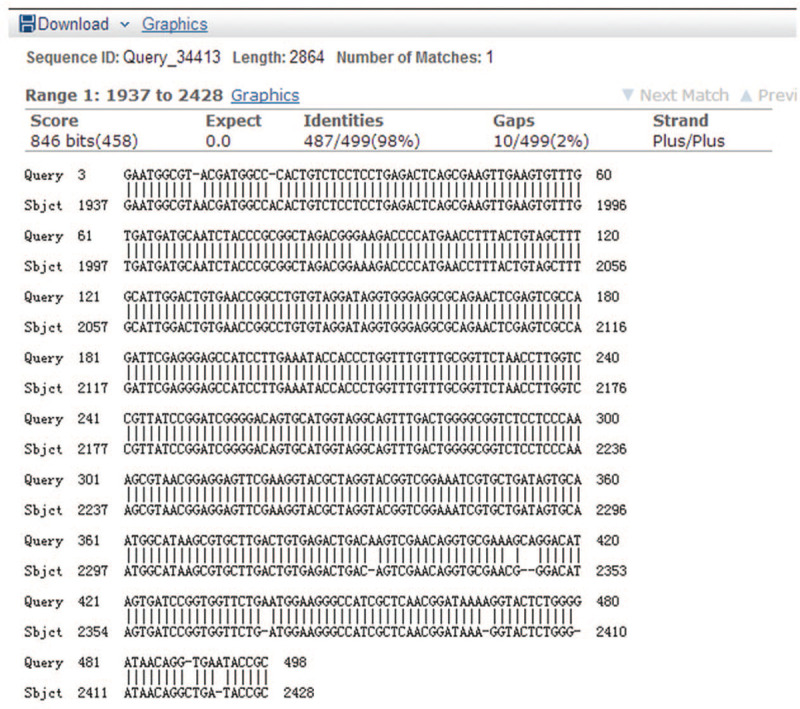
Erythromycin 23S rRNA gene A2047G mutation sequencing.

The erythromycin resistance gene test results of 55 strains of *B. pertussis* showed that 27 strains had the 23S rRNA gene A2047G mutation and these strains had an erythromycin MIC> 256 mg/L. Their MIC values were between 0.016 to 0.094 mg/L. See Figure 2 for the mutation sequencing.

## 4 Discussion

*B. pertussis* can produce a variety of virulence factors, which have been clearly identified as pertussis toxin, pertactin, filamentous hemagglutinin, lectin (fimbriae), and lipid oligosaccharides. Other virulence-related proteins of *B. pertussis* can activate the body's immune response, so it is a candidate vaccine antigen component. After extensive vaccine coverage in many countries around the world, the incidence of the disease has risen again after maintaining a low level for many years, called “resurgence of pertussis”. This phenomenon may be related to the following factors: optimization of diagnostic criteria, laboratory diagnosis with improved methods, and the rising morbidity of older children and adults. The immunization effect of pertussis vaccine, the adaptability of the pertussis vaccine, the decline of vaccine efficacy, and the decline of population immunity have been evaluated imperfectly in the past.^[18]^

The pathogen detection methods of *B. pertussis* mainly include PCR, serological detection of specific antibodies and bacterial culture. PCR detection is simple, time-consuming, highly sensitive, and specific and is recommended as a routine detection method for pertussis. However, this method has some disadvantages, such as false-positives and easy cross-contamination of samples.^[10]^ The serological detection method is simple and feasible, with relatively accurate results and is often used for epidemiological investigation, but it is not conducive to an early diagnosis.^[19,20]^ Bacterial culture is the gold standard for the diagnosis of pertussis. Due to the strict cultural conditions, it is not suitable for early and rapid clinical diagnosis. However, to study the drug resistance of strains, culture is an irreplaceable key step. In this study, 351 children with suspected pertussis were subjected to nasopharyngeal swab bacterial culture, and the positive rate was 16.8%, which was higher than that in other domestic reports.^[11,21]^ The positive detection rate of bacterial culture is related to many factors, such as the location of the sample collection, material, technique, and transportation, and the culture conditions of specimen collection. In this study, we also analyzed the clinical characteristics of these children with suspected pertussis and showed that the age distribution of children with pertussis ranged from 1 month to 7 years old, and the children <6 months old accounted for 50.7%, which is consistent with previous reports.^[21]^ The reasons for the frequent occurrence in this age group may be related to not reaching the age of pertussis vaccination or not completing the whole course of vaccination. We observed that the onset of pertussis in children occurred throughout the year, mostly in the autumn, accounting for 39.4%, and up to 20.4% in August, which is consistent with our previous studies.^[14]^ Some studies have reported that pertussis is more common in the spring and summer, with the highest incidence from April to June.^[22,23]^ This suggests that the incidence of *B. pertussis* infection has regional characteristics. Our study found that the incidence of spastic cough in children with pertussis was more common than that in children without pertussis; the incidence of cyanosis with a history of pertussis vaccination was less than among those without a history of vaccination (*P* < .05), suggesting that after vaccination nearly half of the children can still be infected, but their symptom severity will be reduced (our statistics are only based on the medical history provided by the family of the patients. The family often fails to provide the vaccination registration manual to determine whether they have completed the full vaccination). Our data showed that 50.2% of the children with pertussis had family members with respiratory symptoms before the onset of the disease. However, whether these family members had pertussis was not tested. It has been reported that the confirmed cases of pertussis are clustered in the family, and adults to infants are the main mode of transmission. Thus, parents are the main source of infection for infants and young children with pertussis,^[24]^ which is a problem that needs further attention to our research.

With the increased use of antibiotics worldwide, increasing numbers of bacteria have developed resistance problems, and the treatment of pertussis also faces this serious challenge. Since 1994, there have been reports of *B. pertussis* drug resistance in the United States, France^[7,8,17]^ and other countries. In China, drug resistance strains were reported successively in Beijing, Xi’an, Shandong,^[10,21,25]^ and other areas after 2013. Since the emergence of macrolide-resistant pertussis, monitoring the trend of antibiotic resistance in popular strains has become a public health priority. Yang^[10]^ did a very good job. They selected pertussis strains in different periods and observed that the *B. pertussis* isolated in the 1970 s and 2000 to 2008 were sensitive to macrolides, and then during 2013 to 2014, 91.9% (91/99) of isolates were resistant to macrolides, indicating that the sensitivity of the strains circulating in northern China to macrolide antibiotics has changed over time. The primary change was from macrolide sensitivity to macrolide resistance. Our province is located in southern China, and in this study, 27 (27/55) of our 55 strains, accounting for 49.1%, showed drug resistance, which is still relatively low compared with the reports from northern China. To avoid immune suppression, *B. pertussis* strain will adapt to epidemic changes. In European and American countries, *B. pertussis* epidemic strains with different genotypes were successively isolated (prn2, ptxA1, ptxP3, or prn*-*).^[26–31]^ The erythromycin-resistant strain of *B. pertussis* was first reported in China in 2012, and subsequent reports in several regions indicated that drug resistance was very common in China.^[10,21,32]^ prn1 ptxA1/ptxP1 was the dominant genotype of the virus strain circulating in China, which was different from the genotype of the current epidemic strain circulating in Europe and The United States. Whether the difference in genotype affects the drug resistance of the strain is still unclear. At present, the mechanisms of macrolide resistance are mainly studied by mycoplasma and Gram-positive bacteria, and the possible mechanisms include active exclusion, target change (gene mutation or methylation) and drug inactivation.^[33]^ There has been no systematic study on the molecular mechanism of resistance of *B. pertussis* to erythromycin. Previous studies have suggested that the main mechanism of resistance of *B. pertussis* to erythromycin is the mutation of adenine (A) to guanine (G) at site 2047 of *B. pertussis* 23S rRNA. 23S rRNA is ribosomal RNA, and ribosome is the site of protein synthesis in cells. Erythromycin can inhibit protein synthesis by binding to the peptide chain in the region of 23S rRNA. The mutation A2047G was detected in all of our drug-resistant strains, which was the same as that reported by Bartkus.^[13]^ However, this mutation does not necessarily occur in drug-resistant strains in Beijing, China^[10]^ and Iran,^[34]^ so it is speculated that there may be other unknown resistance mechanisms. Wang Z G^[12]^ systematically detected pertussis chromosome related gene changes and drug-resistant genes mediated by mobile elements, but the results did not find other drug resistance molecular characteristics except A2047G mutation of 23S rRNA gene. Other mutation mechanisms need further study. As for whether erythromycin-resistant pertussis strains will be resistant to other macrolide antibiotics, different reports have come to different conclusions.^[10,21,34]^ In our study, 28 of the 55 strains were still sensitive to erythromycin and clindamycin in the MIC range of 0.016 to 0.094 mg/L, 0.019 to 0.75 mg/L. The MIC50/MIC90 of ampicillin, levofloxacin, and SMZ-TMP were 0.125/0.19, 0.38/0.5, and 0.125/0.25 mg/L, respectively. Since there is no international standard for the resistance dose of these antibiotics for *B. pertussis*, the sensitivity of this experiment in vitro still needs clinical observations and study.

Our subjects included children with suspected pertussis, and it is possible we missed some cases with atypical symptoms, such as cases involving neonates, reinfection, and older children. Mostly the population pattern of pertussis infection transmission is from adults to children, but in our country, testing for *B. pertussis* is rarely performed in adults. Vittucci retrospectively investigated 215 infants with clinical symptoms of acute respiratory tract at the age of 3 months, and the positive rate of pertussis PCR detection was as high as 24.7%.^[35]^ Therefore, we believe that more research is needed to truly reflect the prevalence of the disease in children of different ages. As early as 2006, the United States formulated policies to expand pertussis vaccination, gradually covering young people, nurses, 19 to 64-year-old adults, and pregnant women.^[36]^ Combined with China's national conditions, since in recent years it has also been a peak period of baby births after the opening of the second child policy, so we also look forward to the implementation of this vaccination policy in our country, as well as routine laboratory diagnosis of *B. pertussis* in hospitals.

Pertussis is an acute infectious disease that causes severe respiratory symptoms in small infants. Epidemiological survey data may underestimate its actual prevalence. At present, the vaccination coverage in our country is still limited to infants and young children. We hope that our research can promote the formulation of improved public health strategies.

## Author contributions

**Conceptualization:** Jun Zou.

**Data curation:** Xiaojuan Lin, Lijun Li.

**Formal analysis:** Xiaojuan Lin, Kaihu Yao.

**Funding acquisition:** Lili Zhong.

**Investigation:** Jun Zou.

**Methodology:** Jun Zou, Kaihu Yao, Lijun Li.

**Project administration:** Xiaojuan Lin, Jun Zou, Lili Zhong.

**Resources:** Lili Zhong.

**Software:** Kaihu Yao.

**Supervision:** Lili Zhong.

**Writing – original draft:** Kaihu Yao.

**Writing – review & editing:** Lili Zhong.

## Abbreviations

Abbreviations: *B. pertussis* = *Bordetella pertussis*, MIC = minimum antibiotic concentration, qPCR = fluorescent quantitative polymerase chain reaction, SMZ-TMP = sulfamethoxazole-trimethoprim.
